# Mechano-chemistry of human femoral diaphysis revealed by correlative Brillouin–Raman microspectroscopy

**DOI:** 10.1038/s41598-020-74330-3

**Published:** 2020-10-15

**Authors:** M. A. Cardinali, M. Govoni, D. Dallari, S. Caponi, D. Fioretto, A. Morresi

**Affiliations:** 1 Department of Physics and Geology, University of Perugia, 06123 Perugia, Italy; 2 Reconstructive Orthopaedic Surgery and Innovative Techniques – Musculoskeletal Tissue Bank, IRCCS Istituto Ortopedico Rizzoli, 40136 Bologna, Italy; 3 Istituto Officina Dei Materiali, National Research Council (IOM-CNR), Unit of Perugia, c/o Department of Physics and Geology, University of Perugia, Via A. Pascoli, 06123 Perugia, Italy; 4 Department of Chemistry, Biology and Biotechnology, University of Perugia, 06123 Perugia, Italy

**Keywords:** Raman spectroscopy, Confocal microscopy, Bone, Supramolecular assembly

## Abstract

Brillouin–Raman microspectroscopy is presented as an innovative label-free all-optical investigation approachable to characterize the chemical composition and the mechanical properties of human tissues at micrometric resolution. Brillouin maps unveil mechanical heterogeneities in a human femoral diaphysis, showing a ubiquitous co-existence of hard and soft components, even in the most compact sections. The novel correlative analysis of Brillouin and Raman maps shows that the relative intensity of Brillouin peaks is a good proxy for the fraction of mineralized fibers and that the stiffness (longitudinal elastic modulus) of the hard component is linearly dependent on the hydroxyapatite concentration. For the soft component, a gradient of composition is found, ranging from an abundance of proteins in the more compact, external, bone to abundance of lipids, carotenoids, and heme groups approaching the trabecular, inner, part of the diaphysis. This work unveils the strong potential of correlative mechano-chemical characterization of human tissues at a micrometric resolution for both fundamental and translational research.

## Introduction

Bone is a specialized connective tissue, assigned to provide physical support to the body and to allow, together with muscles and ligaments, its effective motion. Understanding the biomechanical properties of this tissue is crucial, not only to figure out how it can resist compressive stresses, deformations or fractures but also to design novel micro-structured scaffolds with increasingly suitable characteristics for transplanting. Numerous studies have already examined the bone-mineral substance in-depth, underlining the necessity to distinguish different levels of tissue organization from nanoscopic to the macroscopic scale. Due to the complex hierarchical structure of bone, giving a rationale of the microscopic origin of its mechanical properties is a challenging issue^1^.

Mechanical properties of bones are usually studied in vivo by means of quantitative ultrasound. This technique gives global information on bone elasticity but does not allow one to investigate the microscale level because of the limited spatial resolution. Recently, a new method has been proposed to measure the mechanical properties of biological tissues, based on Brillouin micro-spectroscopy^2–7^ The technique is based on the inelastic scattering of light from thermally activated acoustic waves propagating through the sample at GHz frequencies. Based on the light-scattering process, it has the great advantage of being non-destructive and contactless, since it does not need external probes but light. Moreover, thanks to its implementation on top of a confocal microscope, it can reach a micrometric spatial resolution. Brillouin spectroscopy has been already applied to the cortical bovine bone by Sakamoto et al.^8^ to evaluate the bone tissue repairing also in correlation with radiography^9,10^, in porcine articular cartilage^11^, and in the human head of femur by some of us^12^. Here, we propose a synergic integration of Brillouin and Raman microscospectroscopy (BRaMS) by means of a recently developed experimental set-up^13,14^ that is able to get a non-invasive complete assessment of bone biomechanics and biochemistry. Chemical properties of bones have been already profusely investigated by Raman spectroscopy to evaluate bone quality^15–19^, age-related changes, the development of orthopedic diseases, the effect of drugs and medical treatments as well as the correct integration of biomimetic prostheses^17,19–22^. The correlative combination of micro-Brillouin and micro-Raman techniques is here developed to highlight the relationship between composition and mechanical properties of a whole section of human femoral diaphysis, including the external circumferential lamellae, the osteonal region, and the trabecular-like core.

We demonstrate for the first time the coexistence of hard and soft constituents at the micrometric scale through the whole femoral section and we give a method for estimating their relative fractions and the global filling fraction within the bone. Moreover, we highlight how the modulation in local stiffness correlates with chemical composition. Our results show the potential of the integrated use of BRaMS, paving the way for its use in translational research, such as in the early diagnosis of bone diseases as well as in developing innovative scaffolds. In fact, at present, most cases of skeleton disorders depending on primary and idiopathic osteoporosis, Paget’s disease, or bone malignancies, are ascertained only in the advanced stages, when evident pain symptomatology forces the patient to request a medical intervention^23^. Early identification of asymptomatic bone degeneration could be fundamental both for the management of the disease and for the containment of the relative health costs. To this respect, the sensitivity of Brillouin spectra to micro-mechanical properties of the bone reported in the present work suggests Brillouin spectroscopy to be implemented in vivo, e.g. during surgery or in endoscopic applications, together with the already experienced Raman spectroscopy^24^ to discriminate tissue types, as well as diseases, directly in the region of interest within the human body.

## Results and Discussion

**Figure 1:**
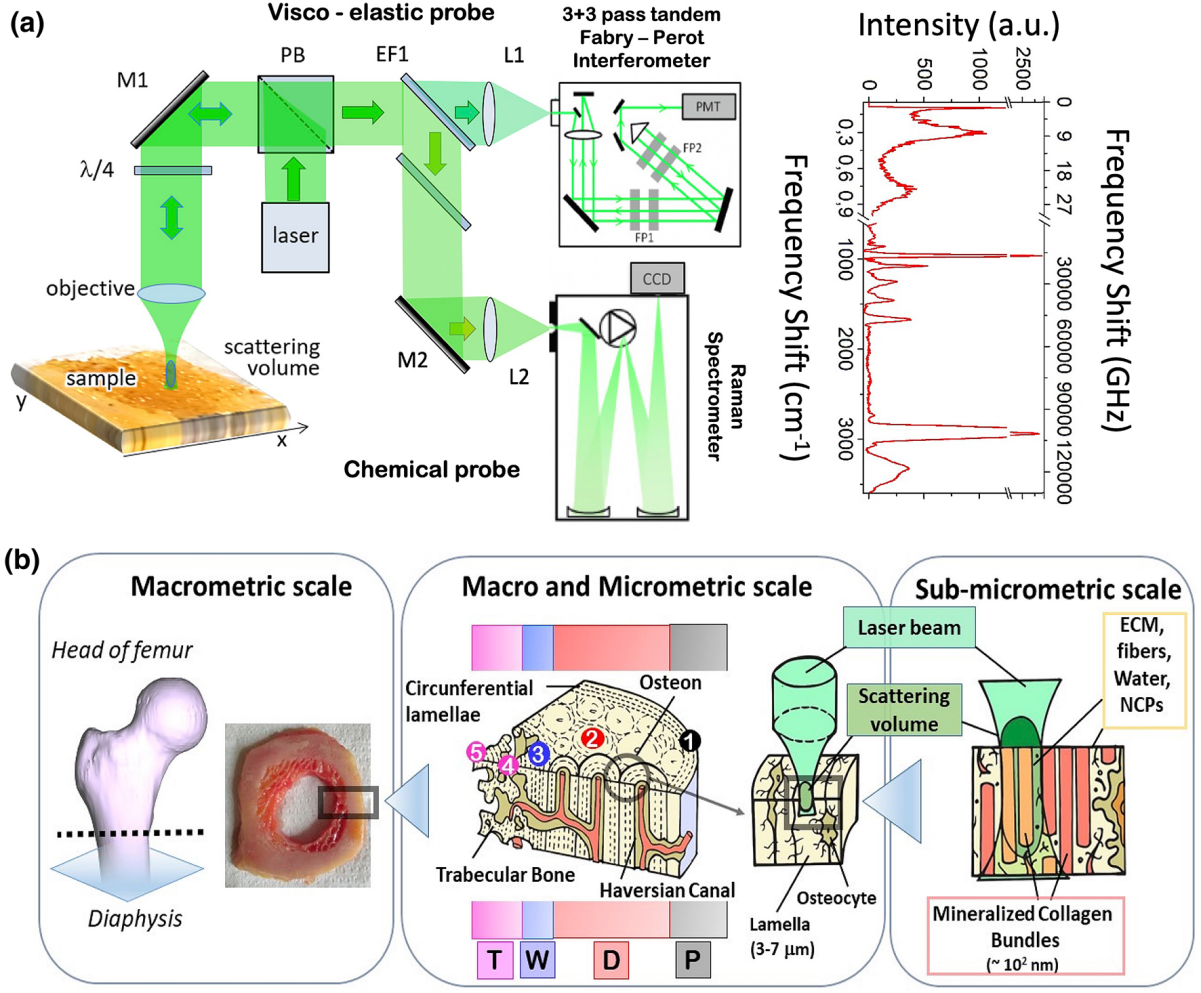
Structure of the sample and scattering geometry. (**a**) Brillouin–Raman microspectroscopy (BRaMS) set-up (left side)^13^ and typical joint Brillouin and Raman spectra (right side) obtained from one single scattering volume of the bone section (see “Methods” for more details). The sample is on an xyz translation stage for mapping purposes. (**b**) Schematic representation of sample hierarchical structure. The structure of the femoral ring excised from a proximal diaphysis is divided into four regions (P—periosteal sheath, D—cortical region, W—transition region, T—trabecular region) going from the periosteal to the inner region. For each measurement, the laser beam enlightens a small surface with a diameter of about 2 μm, which corresponds to a small portion of each lamella composed by mineralized collagen bundles and the not-ordered phase of the extracellular matrix, fibrils, and NCPs.

Micro Brillouin–Raman spectra were collected using the experimental set-up sketched in Fig. 1a. A typical spectrum is reported in the same Figure, showing two well defined Brillouin peaks at frequency shifts lower than 1 cm^−1^. This is the evidence of the coexistence of hard and soft components within the investigated scattering volume. The high-frequency side of the spectrum (frequency shift > 600 cm^−1^) reports a pattern of Raman peaks that gives information on the local chemical composition.

**Figure 2:**
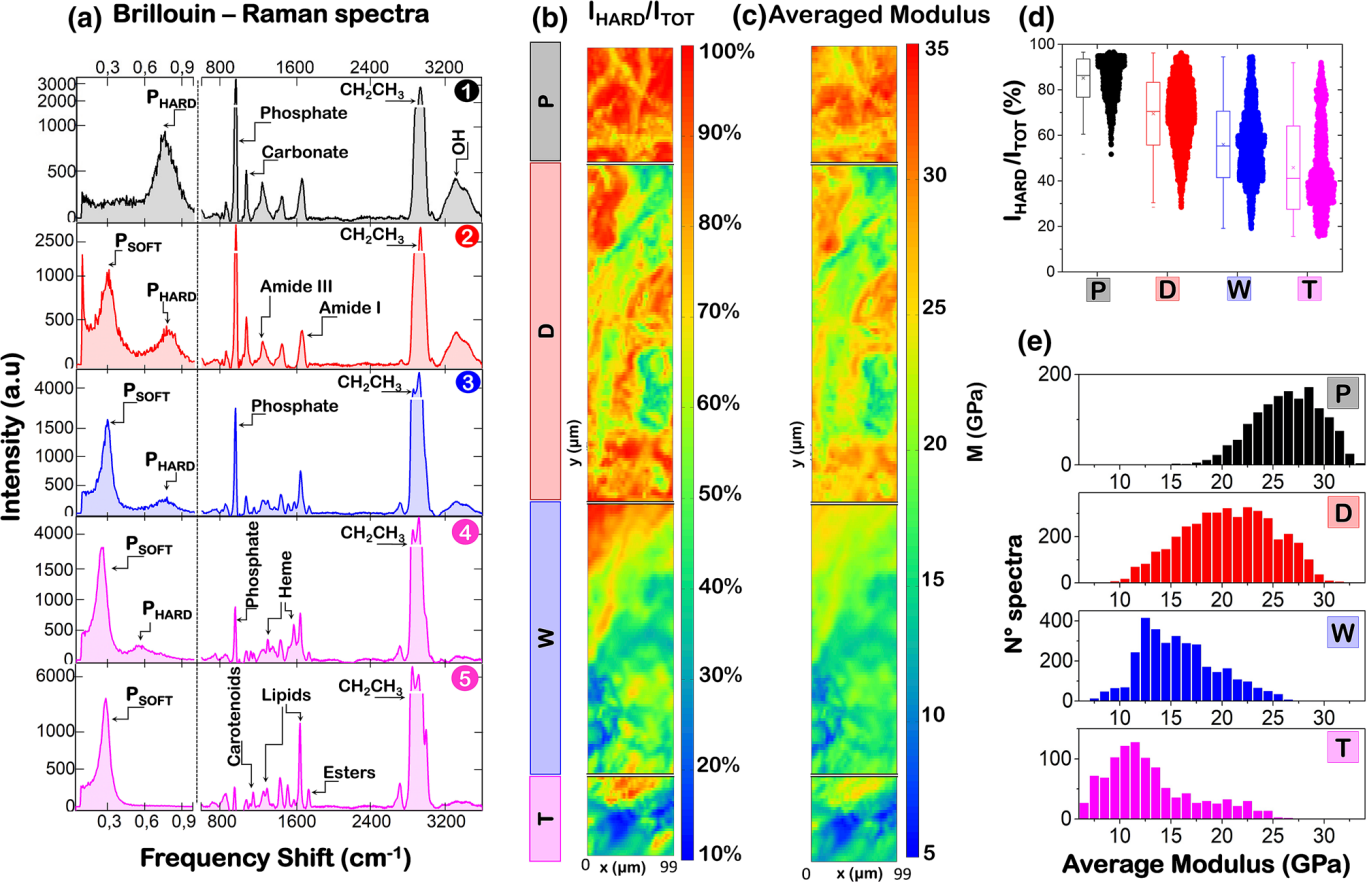
Typical spectra and average mechanical properties. (**a**) Brillouin (frequency shift < 1 cm^−1^) and Raman (frequency shift > 600 cm^−1^) spectra collected from different points (①, ②, ③, ④, and ⑤ in Fig. 1b) of the femur diaphysis, corresponding to the external circumferential lamellae (P- black), the osteonal region (D-red), the transition zone (W-blue) and the trabecular-like core (T-magenta). (**b**) The fraction of hard component obtained as explained in “Methods” by the intensity of Brillouin spectra collected in 2D maps, with 3 μm steps, over the 4 main regions of the diaphysal ring: a 99 × 87 μm^2^ map in T region, 99 × 279 μm^2^ in W, 99 × 348 μm^2^ in D, 99 × 123 μm^2^ in P. (**c**) Point average elastic modulus obtained by frequency shifts and relative abundance of soft and hard Brillouin components. (**d**) Distribution of hard component of Brillouin spectra deduced by the maps in Fig. 2b. (**e**) Distribution of point averaged elastic moduli deduced by the maps in Fig. 2c.

To figure out this complex scenario, Fig. 1b depicts the shape of the micrometric scattering volume, compared with the structure of the bone. The diaphysis ring, excised from the proximal region of a femur (see “Methods” for more details) shows at least four distinct morphologies: the periosteal sheath (P), the cortical region characterized by osteonal structures (D), a transition region (W), and a trabecular-like core (T). From a sub-macroscopic point of view, each of these regions is characterized by mineralized lamellar (hard) structures, with a width of several microns, whose length is organized along the z-axis of the bone to guarantee strength and resistance to mechanical forces. The space between different lamellae is fulfilled by a less organized matrix, which is essential to guarantee the survival of the cellular components of bones. Going deeper into the micro and sub-micro scale, each lamella can be schematized as a composite material constituted by bundles of mineralized collagen fibrils and a not-order (soft) phase: extracellular matrix, thin fibrils of collagen, non-collagenous proteins, and water. Our Brillouin and Raman spectra are sensitive to all of these components, since the confocal microscope sends and collects light from a scattering volume of few cubic micrometers, giving rise to Brillouin and Raman peaks strongly dependent on position, as reported in Fig. 2.

**Figure 3:**
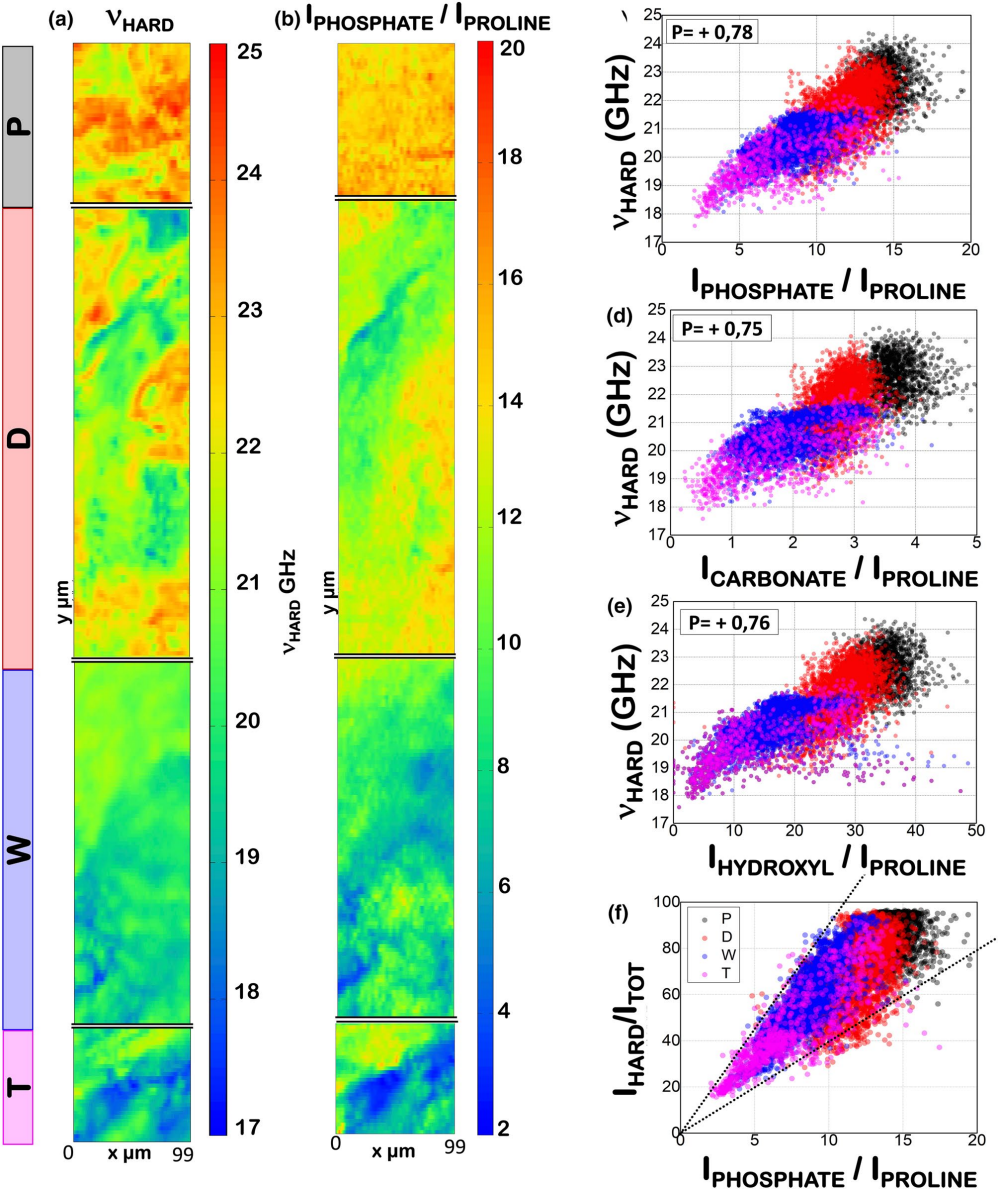
Hard component of the bone and its correlation with hydroxyapatite. (**a**) 2D map of Brillouin frequency shift of the hard component of the bone. (**b**) Intensity of the phosphate Raman peak normalized to the intensity of the proline Raman peak. Correlation between Brillouin frequency shift and Raman peaks of hydroxyapatite components – phosphate, carbonate, and OH groups – are shown in (**c**–**e**), respectively. Pearson correlation coefficients are reported in the insets. (**f**) Relative Brillouin intensity of hard component versus normalized intensity of proline Raman peak. Different colours are used for data obtained from the different regions of the bone outlined in Fig. 1.

**Figure 4:**
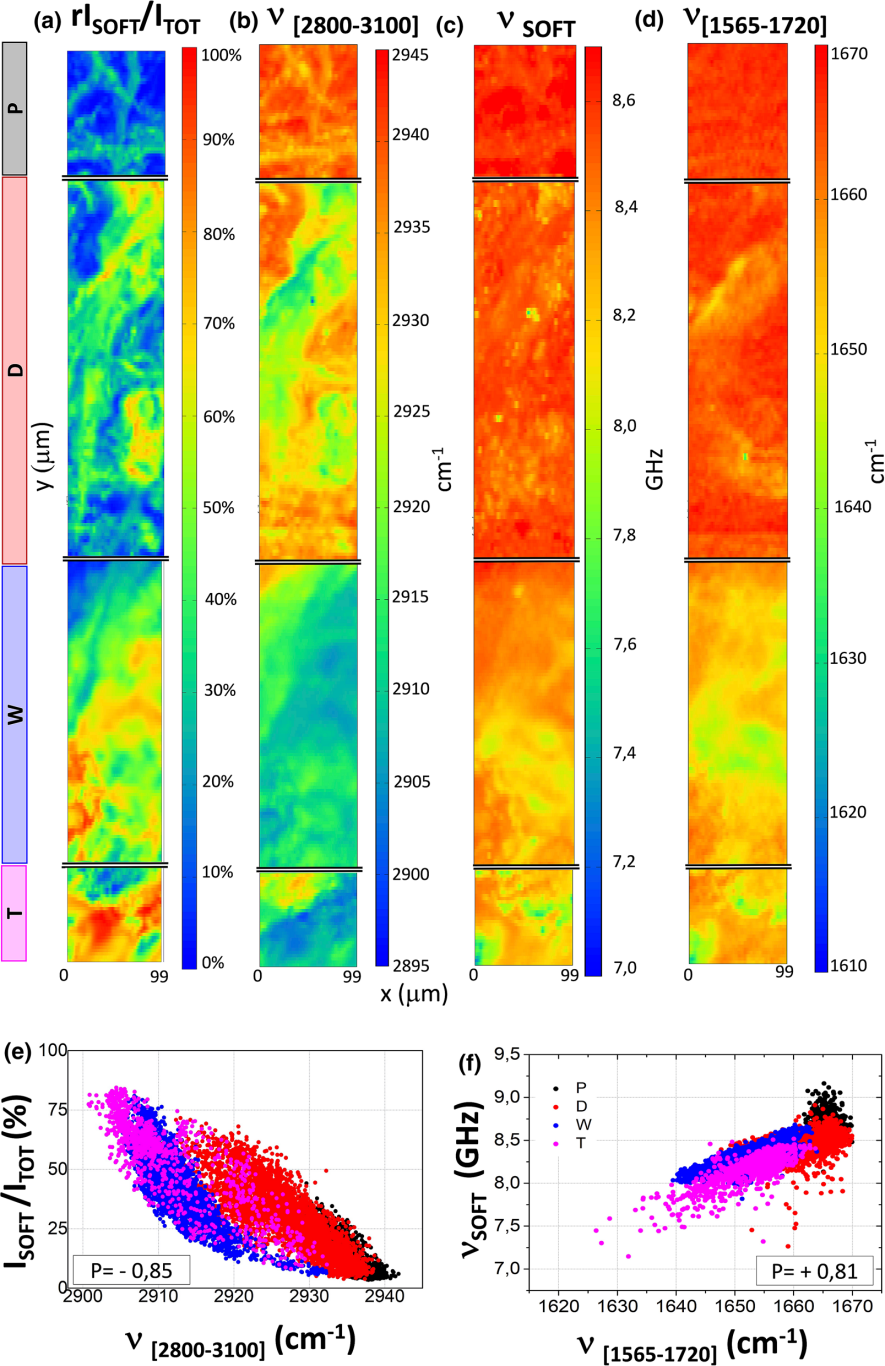
Soft component of the bone and its chemical composition. (**a**) Fraction of soft component of the bone, obtained from the data in Fig. 2b as 1–I_HARD_/I_TOT_. (**b**) Average frequency (first spectral moment) of the CH_2_CH_3_ stretching Raman band in the range 2800—3100 cm^−1^ of Fig. 2a. (**c**) Average frequency of the soft Brillouin peak, P_SOFT_ in Fig. 2a. (**d**) Average frequency of (Heme + Amine I) Raman band in the range 1565—1720 cm^−1^ of Fig. 2a. (**e**) Correlation between relative intensity of Brillouin soft peak and first spectral moment of the CH_2_CH_3_ Raman band in the range 2800—3100 cm^−1^. (**f**) Correlation between Brillouin frequency shift of the soft peak and first spectral moment of (Heme + Amide I) Raman band in the range 1565—1720 cm^−1^. Different colours are used for data obtained from the different regions of the bone outlined in Fig. 1. Pearson correlation coefficients are reported in the insets.

**Figure 5:**
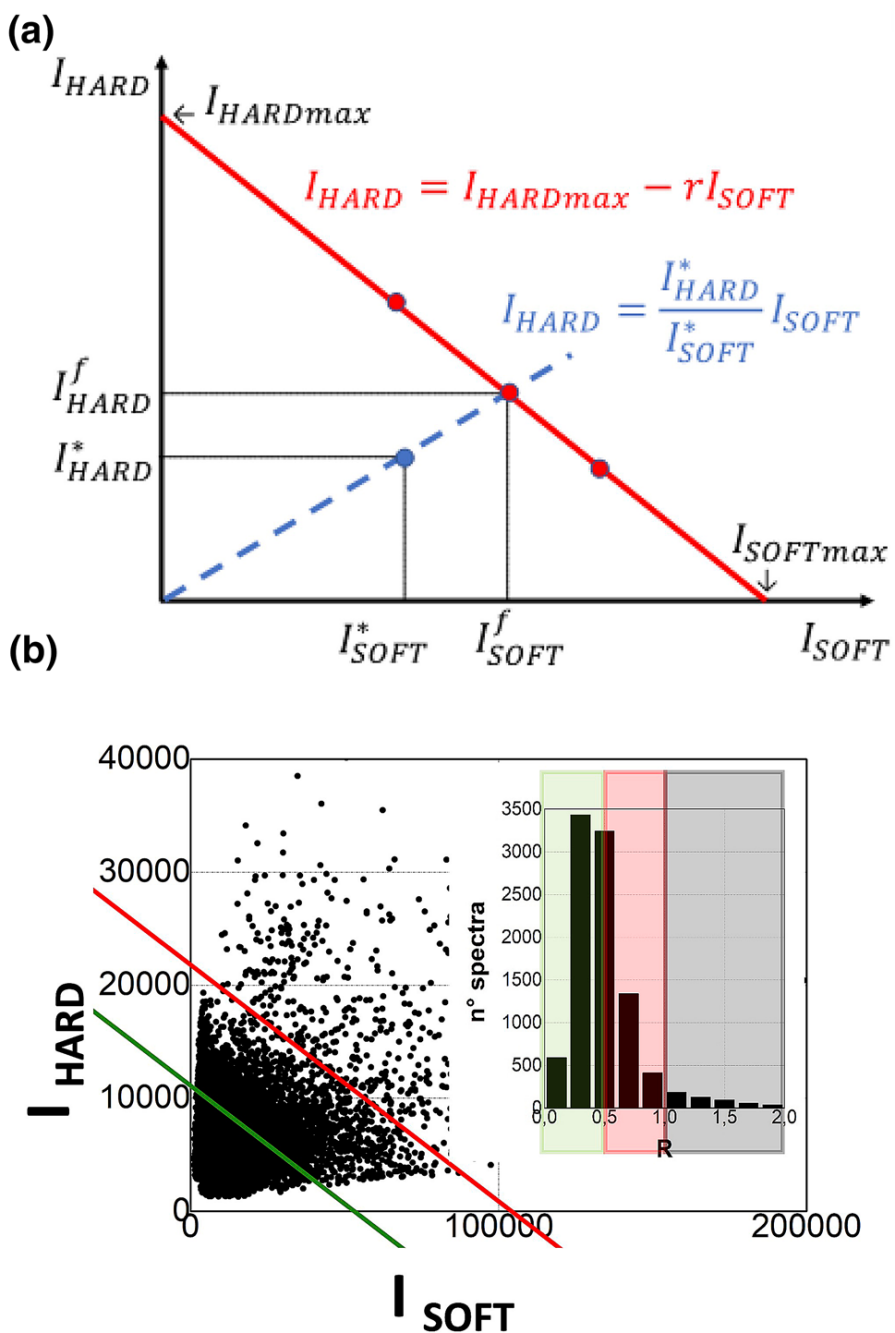
Intensities of Hard and Soft Brillouin peaks of the bone, reported in a Cartesian plane (**a**) can be used to determine the average ratio of squared Pockels coefficients (*r*) and filling factors $R = I_{HARD}^{*} /I_{HARD}^{f}$. (**b**) The whole set of pairs of $I_{SOFT}$ and $I_{HARD}$ Brillouin intensities obtained from our sample (black dots) with the linear interpolation (red line) whose angular coefficient gives *r* = 0.21 . In the inset, the distribution of filling factors obtained by Eq. 3.

Figure 2a shows typical Brillouin (frequency shift < 1 cm^−1^) and Raman (frequency shift > 600 cm^−1^) spectra obtained from the external circumferential lamellae (P- black), the osteonal region (D-red), the transition zone (W-blue) and the trabecular-like core (T-magenta), collected in the five different points of the femur diaphysis, labeled ①, ②, ③, ④, and ⑤ in Fig. 1b. We notice that soft and hard components are present in all regions, giving rise to Brillouin peaks at ~ 0.3 and ~ 0.7 cm^−1^, respectively. The Raman spectra are representative of the characteristic chemical phenotype of the four analysed regions. In fact, while the external circumferential lamellae and the osteonal regions (Fig. 2a, ① and ②) show the typical signals of collagen fibers and hydroxyapatite crystals, the trabecular-like region shows signals from the Heme group, due to abundance of red bone marrow (Fig. 2a, ④), and from lipids and carotenoids, characteristic of the bone marrow adipose component (Fig. 2a, ⑤). In the transition region (Fig. 2a, ③), signals from both the mineralized collagen fibers and the organic part of the bone marrow are present. The extremely heterogeneous mechanical and chemical properties of the sample and their mutual correlations will be appreciated in the maps of Figs.3 and 4. Here we focus on average values of the tissue elastic constants and their distributions along the investigated section, to be compared with the results previously obtained by more traditional mechanical investigations.

### Bimodal Brillouin spectra: evidence of soft and hard components, mixed at micrometric scale in the whole sample

Most of the Brillouin spectra collected through the sample present two peaks: one centred at high frequency (P_HARD,_ in the range 17–25 GHz, i.e. 0.56–0.83 cm^−1^) and the other shifted at lower frequencies (P_SOFT,_ in the range 8–9 GHz, i.e. 0.27–0.3 cm^−1^). The simultaneous detection of two Brillouin peaks in a single spectrum suggests the coexistence of elastically different structures at micrometric length scale^25^. The longitudinal elastic modulus *M* of each component can be obtained from the Brillouin frequency shift *ν* through the relationship:

1 $$M = \frac{\rho }{{n^{2} }}\frac{{\lambda^{2} }}{4}\nu^{2}$$

where *ρ* is the mass density, *n* the refractive index and *λ* the wavelength of the laser source. In the following, we take *ρ* = 2 gr/cm^3^ and *n* = 1.55^26^ and assume a constant ratio *ρ* / *n*^2^ through the whole sample. This approximation has been recently tested in water-collagen mixtures as a function of water content, from the liquid-like condition (similar to our soft component) to the almost dry sample (similar to the hard component of the bone) and found that the change in ratio *ρ* / *n*^2^ is less than 1% across the whole concentration range^27^. Furthermore, neglecting additional heterogeneity induced by local changes of composition is here assumed as a reasonable approximation, as recently found in different tissues^28^. We thus obtain the modulus of the hard component *M*_H_ to be in the range 17–37 GPa, and of the soft component *M*_S,_ in the range 3.8–4.8 GPa. Considering the structural organization of bones at micrometric scale (Fig. 2b), the hard component can be tentatively attributed to an ordered phase of tight bundles of (more or less) mineralized collagen fibrils which principally contributes to the stiffness and mechanical resistance, and the soft component to a not-ordered phase composed by poorly mineralized collagen fibers, non-collagenous proteins, glycosaminoglycans (GAGs), water, etc..

The simultaneous characterization of hard and soft components of the bone at micrometric scale is a novelty not only with respect to more usual investigation methods but also with respect to previous Brillouin Scattering studies^8,9^. It is likely made possible by our choice of relatively thick sections and minimal sample pre-treatment. The coexistence of Brillouin signals from both hard and soft components was anticipated by some of us on a different sample (human femoral head) treated with a different protocol (fixation in paraformaldehyde and storage in ethanol)^12^. We can anticipate that this is a robust experimental result, confirmed by all samples until now analyzed by our technique. In the next paragraphs, we will show that the bimodal Brillouin spectrum enables one to estimate not only the stiffness but also the local volume fraction of soft and hand components and the correlative Raman analysis gives their molecular composition. This is a chief result of our work since the micrometric intercalation of soft and hard components leads to important implications in the bone tissue mechanical performances and functionality. In fact, the micrometric lacunar-canalicular structure here revealed has been suggested as the most important porosity length scale for poroelasticity and mechanosensory effects in bones^29^. The movement of the interstitial bone fluid in pores guarantees transports nutrients and the removing of cell waste and is the basic mechanism for the activation of mechanotransduction and bone regeneration^30^.

### Point-average elastic modulus and comparison with previous investigations

Brillouin and Raman spectra were collected in 2D maps, with 3 μm steps, as described in Fig. 2. The intensity of Brillouin peaks has been used to estimate the volume fraction of the hard component of the bone within the scattering volume ($V_{H}$), for each point of the map, by the relationship $V_{H} = I_{HARD} /{ }I_{TOT}$ as described in “Methods”. The 2D map of $I_{HARD} /{ }I_{TOT}$ is reported in Fig. 2b and its statistical distribution in Fig. 2d showing, as expected, an average value that progressively decreases when moving from the outer to the inner part of the diaphysis, together with an increase of the mechanical inhomogeneity (width of the distribution). The elastic modulus considerably changes in different regions of the diaphysis. In fact, not only the volume fraction of hard and soft components changes but also their frequency shifts, revealing that the stiffnesses of the two phases are modulated through the tissue. The spatial behaviour of the elastic moduli of hard and soft components are separately analysed in Figs. 3 and 4, while here (Fig.2c,e) we report the average modulus *M* calculated, for each point of the map, through the Voigt model, i.e. $M = V_{H} M_{H} + \left( {1 - V_{H} } \right)M_{S}$, which has been recently found to be valid also in case of isotropic gelatin samples in a wide range of water concentrations^27^. The results of Fig. 2 can be compared with those previously obtained by different techniques at different spatial scales.

To this respect, it is interesting to notice that both nano-indentation and scanning acoustic microscopy (SAM) have shown similar values for the elastic modulus of both cortical and trabecular bone, though the Young modulus was measured in that case instead of longitudinal modulus. In fact, for our solid sample, the viscoelastic effects are less severe than for liquids and gels, so that the values of the elastic moduli measured by Brillouin Scattering are expected to be of the same order of magnitude of those measured by quasi static techniques. In particular, an average value of 20.55 GPa by SAM and 23.45 GPa by nano-indentation was found on the longitudinal direction of the femoral diaphysis by Turner et al.^31^ Other studies reported slightly different values of the Young modulus going from the femoral neck region to the mid-shaft diaphysis: in particular, nano-indentation measured 15.8 GPa and 17.5 GPa for the osteonal and the interferential lamellae in the femoral neck and 19.1 GPa and 21.2 GPa for osteonal and interferential lamellae in the femoral mid-diaphysis. For the femoral trabecular bone, averaged values of 17.50 GPa and 18.14 GPa were measured by SAM and by nano-indentation, respectively, in the distal diaphysis^32^. On the other hand, Chevalier et al.^33^ reported higher values, going from 19.6 to 21.8 GPa on the dried trabecular bone excised from three different femoral heads. Results obtained by our group on the femoral diaphysis revealed an averaged longitudinal elastic modulus of 26.41 GPa in the periosteal region, 20.68 GPa in the osteonal region, 15.83 GPa in the transition zone, and 13.05 GPa in the trabecular bone core^12^. As a whole, we can say that the average values of our results are consistent with previous ones. The novelty here is that we are able to map the tissue on a micrometric scale and distinguish the soft and hard components.

### Hard component and its correlation with hydroxyapatite content

Figure 3a shows maps of the frequency shift *ν*_HARD_ of the hard component of the bone, obtained from the high-frequency Brillouin peaks (P_HARD_ in Fig. 2a). Consistent with the structure of the bone, it can be seen that the highest frequency shifts (hardest portions of the bone) are mainly located within the external circumferential lamellae and the osteonal region, and that the lowest frequency shifts (softest portions) are mainly within the transition zone and the trabecular-like core. It is important to note that the factor 1.4 found between maximum and minimum values of $\nu_{HARD}$, indeed corresponds to a considerable factor 2 between upper and lower bounds of the elastic modulus (see Eq. (1) and Fig. 2e). Moreover, although there is a general trend from the more rigid external part to the softer internal one, the distribution of frequencies is quite heterogeneous within each region. In this condition, the correlative use of Raman spectra is precious to ascribe the microscopic origin of both the general trend and the local modulation of hardness.

The basic component giving mechanical resistance to the bone is the mineralized collagen fiber which is composed of fibrils of type I collagen and aggregates of carbonate hydroxyapatite nanocrystals. A correlation between hardness, revealed by the frequency of Brillouin peaks, and hydroxyapatite content, revealed by the intensity of the Raman peaks of phosphate (959 cm^−1^) carbonate (1070 cm^−1^) and OH (3400 cm^−1^) groups, should be expected along the tissue. Raman intensities are normalized to a representative Raman peak, the proline stretching peak of the collagen fibers, in order to probe variations of mineralization with respect to the total collagen content^17,34,35^. Figure 3b shows the 2D map of the phosphate Raman signal. Visual comparison of Fig. 3a with Fig. 3b already suggests a good correlation between the frequency position of the hard component $\nu_{HARD}$ and the presence of phosphate groups. The existence of such a correlation is better visualized by the scatter plot of Fig. 3c, which reports $\nu_{HARD}$ of the four investigated regions as a function of the normalized intensity of the phosphate peak. Similar correlations can be seen in Figs. 3d and 3e for the relative variation of $\nu_{HARD}$ as a function of the intensity of the other two spectroscopic signatures of the mineralized component: the carbonate and OH groups, respectively. These results suggest that the factor two modulation in the stiffness of the micrometric hard component of the bone is due to a variation in the hydroxyapatite content. Moreover, we can see that on average the inner part of the bone is softer than the outer one not only for the larger amount of soft matter (Fig. 2.b) but also for a lower degree of mineralization of collagen fibrils.

We have proposed the normalized intensity of Brillouin peaks $I_{HARD} /{ }I_{TOT}$ as a measure of the hard fraction of the bone within the investigated volume. In Fig. 3f we compare this parameter with the relative fraction of hydroxyapatite deduced by the intensity of Raman peaks, the different colors indicating the four different investigated regions. A peculiar behaviour is obtained, where almost all points are within a triangular region delimited by two straight lines passing through the origin of the axes, with angular coefficients that are one double the other. This singular trend can be understood in terms of different cross-sections for the phosphate Raman mode (ν_1_PO_4_^3−^), depending on the polarization of light with respect to the orientation of the hydroxyapatite crystal^22,36,37^. In fact, previous studies revealed that a factor almost two does exist for the scattered intensity in case of light polarized parallel or orthogonal to the mineralized collagen fibres in bovine bone diaphysis^38^. Due to the different orientation of fibers in different regions of our sample, it seems thus reasonable to have a factor two in the distribution of scattered intensities for a given mineral fraction. This interesting result reinforces the validity of the procedure here adopted for the treatment of Brillouin and Raman data and confirms that the relative intensity of Brillouin peaks is indeed a good proxy for the evaluation of the relative scattering volumes in our biphasic system.

### Soft component and its correlation with lipids and Heme abundance

Figure 4a shows the fraction of soft component deduced from the relative intensity of P_SOFT_ in our four mapped regions. This contribution is particularly expressed in the transition region (W) and in the trabecular-like zone (T), where it reaches the highest values in terms of relative intensity. Comparing Fig. 4a with Fig. 4b it can be seen that the fraction of soft component correlates well with the abundance of lipids within the scattering volume. In fact, an increasing abundance of lipids gives rise to a shift towards lower frequencies of the average position of the CH_2_ CH_3_ Raman stretching band located between 2800 and 3100 cm^−1^ (Fig. 4b), due to a relative increase of the lipids peaks, i.e. the CH_2_ symmetric and antisymmetric stretching modes located at 2855 and 2880 cm^−1^^39^, with respect to the CH_3_ stretching peak at 2935 cm^−1^, mainly attributed to proteins. Variations in the relative intensity of these components are directly visible from the raw data recorded within the trabecular-like core reported in Fig. 2a. The existence of such a correlation between the soft component and lipid abundance is highlighted in the scatter plot of Fig. 4e.

Figure 4c shows the Brillouin frequency shift *ν*_SOFT_ of the peak P_SOFT_ within the four mapped regions. A general reduction of P_SOFT_ frequency shift is found moving from the external towards the internal side of the bone, together with a quite mild local modulation, more evident in the transition and trabecular zones. These fluctuations are correlated with the intensity of the Raman signal generated by Heme ring vibrations, typical of blood and red marrow cells, reported in Fig. 4d. The existence of such a correlation is confirmed by the scatter plot of Fig. 4f, reporting the values of *ν*_SOFT_ detected through the whole sample (different colours for the different zones) vs. the average Raman frequency in the range 1565 and 1720 cm^−1^. Since Heme and Amide I peaks are included within the large spectral feature between 1565 and 1720 cm^−1^ (see Fig. 2a), their relative contributions have been estimated from the average frequency (first spectral moment) of this spectral region. In particular, a reduction of the average frequency occurs when the Heme ring vibrations are more intense with respect to the Amide I signals. Notice that the periosteal sheath (P—black), the cortical region zones (D—red) are, as expected, Heme-free, and that the Brillouin frequency reduces for increasing Heme concentration in the transition region (W—blue), and a trabecular-like core (T—magenta). We can thus reasonably recognize that proteins dominate the composition of the soft fraction of the external circumferential lamellae and that a more heterogeneous scenario is present in the osteonal and transition zones, with a progressive shift towards higher lipid and heme content penetrating inside the bone, corresponding to a progressive softening of the soft fraction of the bone. These results reinforce the idea that we can detect the cellular components of the tissue, which are necessarily confined into the soft, not-ordered phase of extracellular matrix and not-mineralized collagen fibers to survive. These cellular components include both the osteocytes and the osteoblasts, which remain embedded into the osteonal region after the mineralization of the matrix, but also cells that constitute the bone red marrow enclosed among the trabeculae.

## Conclusions

For the first time, the mechanical biphasic nature of bones has been successfully revealed by Brillouin imaging, evidencing the ubiquitous co-existence of two elastically different structures at the micrometric length scale in the whole range passing from the external circumferential lamellae to the trabecular-like core of the diaphysis. This evidence gives support to the poroelastic treatment of bone’s elasticity and allows one to get local information on both fractions of soft and hard components and their local elastic moduli. The micrometric scale mapped by Brillouin micro-spectroscopy is that of the lacunar-canalicular porosity, the most important porosity scale in terms of mechanical and mechanosensory effect in bones, with great potential in terms of characterization of site-specific textures for future tissue engineering strategies. In addition, a correlative analysis of Brillouin and Raman maps has been here introduced, associating chemical composition to the mechanical properties of the tissue. This morpho-chemo-mechanical mapping of bones has strong potential in revealing the development of pathologies and their correlation with elastic properties and chemical composition at the micrometric scale.

## Methods

### Sample collection

The human bone sample was collected from a cadaveric donor (male, 60 years of age) without a history of bone disorders by the Muskoloskeletal Tissue Bank of IRCCS Istituto Ortopedico Rizzoli (Bologna, Italy). In this study, only a specimen not suitable for transplantation and considered as waste material was used, according to the Italian law and National Transplantation Center’s guidelines. The femoral sample was obtained by retrieving a 5 mm thick ring from the lesser trochanter, about 2 cm below the femoral epiphysis. Then, the tissue was washed with sterile physiological saline solution and stored at -80 °C. Besides, its safety was monitored by specific microbiological tests before performing spectroscopic analyses.

Before starting measurements, the sample was thawed out and washed twice with distilled water to remove the residual blood. After that, it was left to dry at room temperature for about 24 h. Water content was monitored by the intensity of the –OH stretching signal in the region between 3150 and 3600 cm^−1^, till when it was found to stabilize in time.

### Sample structure

A photo of our sample is reported in Fig. 1b, together with a series of cartoons illustrating the hierarchy of structures from the macro to the sub-micron level, compared with the scattering volume of our setup. Concerning lamellar bone, the first hierarchical model consisting of 7 different structural levels, identified in 1986 by Weiner and Wagner^40^, has been further extended by Reznikov et al.^41^ by revealing a fractal-like organization in which each structural level presents two different phases: a predominant ordered component, composed by arrays of mineralized collagen fibrils organized in precisely oriented patterns, and a disordered one, composed by a matrix with poorly oriented collagen fibers and a great amount of non-collagenous proteins (NCPs), proteoglycans and water. At *sub-micrometric scale*, the basic component of bone substance is the mineralized collagen fiber, which is composed of fibrils of type I collagen and aggregates of carbonate hydroxyapatite nanocrystals^42^. Groups of mineralized collagen fibers are adjusted in wide arrays, characteristic of the ordered phase, with a diameter variable from less than a micron to several microns to form bundles. The space between different bundles is fulfilled by the disordered phase, which contains the osteocytes and their extracellular processes in precise niches defined lacunae and canaliculi respectively. At the *microscopic level*, each lamella (3-7microns) consists of a series of bundles sheets, which constitute the ordered phase, and a disordered component in which are present canaliculi perpendicularly aligned on respect to the principal axis of the bone. This is the structural level mapped by our BRaMS.

### Experimental setup (Fig. 1a)

Light from a 532-nm single-mode solid-state laser source is reflected by a polarizing beam splitter (PB) into a 20 × microscope objective lens for imaging, with a numerical aperture of 0.42, lateral spatial resolution of about 2 μm in diameter and about 15 μm axial resolution, measured from the behavior of the intensity of Brillouin peaks through the air-sample interface. Laser power was about 7 mW in the sample. The backscattered light is split in frequency and direction by an edge filter (EF1), so that the high frequency-shift Stokes component (> 30 cm^−1^) is sent to the Horiba iHR320 Triax Raman monochromator and the low frequency-shift (< 30 cm^−1^), the quasi-elastic, and the anti-Stokes component are sent to the multi-pass tandem Fabry–Perot interferometer (T-FP2). Acquisition time was about 75 s for each point. The sample is mounted on an xyz translation stage for mapping purposes. More details on this recently developed setup are reported in Ref.^13^.

### Data elaboration

#### Brillouin spectroscopy

Brillouin spectroscopy deals with light inelastically scattered by acoustic modes thermally activated within the sample. The frequency shift of Brillouin peaks with respect to the laser source, *ν*, gives the longitudinal elastic modulus *M* of the medium through Eq. (1). Intensity and frequency shift of Brillouin peaks were calculated through spectral moments as described in Ref.^12^, by averaging Stokes and anti-Stokes contributions. In particular, we estimated the average frequency shift of each peak through the calculation of the first spectral^43,44^, i.e. $\bar{v} = \sum\nolimits_{i} {I_{i} v_{i} } /\sum\nolimits_{i} {I_{i} },$ where the index *i* spans spectral channels in the range 4–13 GHz and 13–32 GHz for the low-frequency (*ν*_SOFT_) and high-frequency (*ν*_HARD_) modes, respectively. The use of spectral moments helps to reduce the error in evaluating the average Brillouin frequency shift in case of a heterogeneous broadening of the peak since it does not rely on the arbitrary choice of a fitting function. By the use of this method, we have generally found robust and reproducible results, with variances smoothly degrading as the square root of the peak area^12^. In the case of micro-structured material, Brillouin spectra can be used not only to characterize the mechanical properties of the different components but also to determine the relative volume fraction occupied by the constituents within the scattering volume. In fact, for any given component the intensity of the Brillouin peak is associated with its volume fraction weighted by the appropriate squared Pockels coefficient^44^. In our biphasic system, we estimated the volume fraction of the hard component of the bone ($V_{H}$) through the relationship:

2 $$V_{H} = I_{HARD} /{ }I_{TOT} = { }I_{HARD} /{ }(I_{HARD} + rI_{SOFT} )$$

where $I_{HARD}$ and $I_{SOFT}$ are the integrated intensities of P_HARD_ and P_SOFT_ Brillouin peaks, respectively, and *r* the ratio between the maximum measurable intensities, $I_{HARDmax}$ and $I_{SOFTmax}$, corresponding to single components filling the whole scattering volume.

Here we introduce a method to estimate the value of *r* in practical cases, based on the representation of the whole set of measured spectra in a Cartesian plane, with $I_{SOFT}$ and $I_{HARD}$ as main axes. In this representation, the points representing spectra obtained from equally filled scattering volumes should fall along the red line in Fig. 5a and few points would be enough to obtain the value of *r*, the angular coefficient of the interpolating straight line. In the less ideal case of partially filled scattering volumes, the intensity of Brillouin peaks would be lower and all points representing measured spectra should fall below the red line in Fig. 5a. It is interesting to notice that in this case, though the measured intensities $I_{SOFT}^{*}$ and $I_{HARD}^{*}$ would be lower than those obtainable from filled scattering volume, say $I_{SOFT}^{f} and I_{HARD}^{f}$, Eq. (2) can still be used to obtain the volume fraction of the hard component and that the filling fraction *R*, i.e. the fraction of the scattering volume filled by the sample—represented by the point ($I_{SOFT}^{*}$,$I_{HARD}^{*}$)—can be obtained by its position in the blue line in Fig. 5a., i.e. by the relationship:

3 $$R = I_{SOFT}^{*} /I_{SOFT}^{f} = I_{HARD}^{*} /I_{HARD}^{f} = \left( {I_{HARD}^{*} + rI_{SOFT}^{*} } \right)/I_{HARDmax}$$

Following this method, in Fig. 5b we have reported the whole set of pairs of Brillouin intensities obtained from our sample. A triangular symmetry is visible, well represented by the red line characterized by the angular coefficient *r* = 0,21. This value can be considered as the average ratio of the squared Pockel coefficient for the hard and soft portions of the sample. It has been used in Eq. (2) to estimate the values of $V_{H}$ reported in Fig. 2b. We notice that the wide scattering of the data in Fig. 5b can be attributed to the roughness of the surface, which is of the order of the length of the scattering volume, or even larger. The distribution of values of the filling fractions calculated from these points through Eq. (3) is reported in the inset of Fig. 5b. It can be seen that the distribution has a maximum roughly corresponding to R = 0.5, which matches the maximum sharpness of the optical image recorded through the microscope. It can also be seen that a few points (< 5%) fall above R = 1. They possibly correspond to conditions where the sample fills more than the scattering volume, so that light is collected also from the tail of the point spread function, close to the optical axis.

#### Raman spectroscopy

Raman spectroscopy deals with light inelastically scattered by inter and intra-molecular vibrational modes thermally activated within the sample. The data analysis was performed using in-house software that eliminates spikes and subtracts the luminescence. The frequency position of each Raman peak is the signature of a chemical bond of a specific molecule, and the intensity is proportional to its concentration multiplied by its optical activity. To eliminate spurious effects related to possible fluctuations of the intensity of the laser source, slight modulations of the scattering volume, etc., intensities of Raman peaks are usually normalized to that of a well-recognised peak. In our case, we chose the intensity of the proline stretching peak, which is well known to give the collagen content^17,33,35^. Conversely, in those cases when we report the average frequency shift of a wide Raman band, we calculate the first spectral moment as described above for the Brillouin spectra, which does not require further normalization procedures.
